# Size effect on contact behavior in DEM simple shear tests

**DOI:** 10.1038/s41598-021-99473-9

**Published:** 2021-10-07

**Authors:** Yao Li, Jiaping Li, Tantan Zhu, Kuan Han

**Affiliations:** Chang’an University, Xi’an, 710064 China

**Keywords:** Civil engineering, Theory and computation

## Abstract

The 2–2.5 times the simulated sand diameter is widely accepted in giving reasonable DEM simulation results for geotechnical testing. However, it neglects the effect of a specimen height to maximum particle diameter ratio in a specific laboratory test, which may lead to a strong stress concentration and flawed simulations. This study compared laboratory simple shear tests with corresponding DEM simulations with different particle sizes. The DEM model used clump rings to simulate physical rings in the test, and decreased the additional stress applied by the widely used wall-type rings. Results showed that (1) DEM models with tested particle size and twofold sand particle size (1D and 2D tests) can better capture the tested stress–strain behavior, volumetric changes, and noncoaxiality, the 4D model has an asymmetrical distribution of contact force and contact number, indicating the specimen is inhomogeneous and has a strong stress concentration. (2) a specimen height to maximum particle diameter ratio smaller than 10 (it is greater than 10 in the ASTM D6528) could provide reasonable macro-meso mechanical behaviors. Similar studies should be carried out after trial tests on determining a reasonable specimen height to maximum particle diameter ratio under the guidance of ASTM D6528.

## Introduction

In geotechnical engineering, it is widely accepted that the size of particles has a significant effect on the shear behavior of sand, and the effect is considered the same important in DEM simulations^1^. Due to large quantities of calculations are involved in simulations with small particle sizes, especially in 3D simulations, most DEM simulations use particles with a larger diameter compared with the sand average diameter (D50). Some researchers stated that particles with 2–2.5 times the material diameter give reasonable results in DEM simulations^2–4^. However, the size effect is completely different in various simulations, for example, in different simulations, DEM models may have different boundaries, particle size, and specimen scales which will dramatically affect the simulated results. Therefore, previous findings about particle size multiplier cannot be directly applied in different models. Instead, the ratio of short boundary to the maximum particle diameter should be used.

In previous DEM studies on simple shear tests, Asadzadeh and Soroush^5^ used 67,000 particles with a uniform diameter of 1.12 mm to simulate the glass beads ranged from 1 to 1.1 mm in a 3D DEM simple shear test with a specimen height of 20 mm and diameter of 70 mm. The simulated particles are generally in the same size as simulated glass beads, and the specimen height to particle diameter ratio is 17.86. Gutierrez and Muftah^6^ used 6556 to 16,190 particles with diameters of 0.3 to 0.8 mm in a 2D DEM simple shear test with a specimen height of 6 mm and diameter of 12 mm. The test height (short boundary) to particle diameter ratio is 7.5 to 20. The particle size is selected based on balancing calculation time and representative element volume, it also mentioned that the particle number should be greater than 5000 to assure a representative element volume in simulating the stress–strain behavior of granular material. Bernhardt et al.^7^ used a 7500 equal number of particles with the diameter of 2.38, 3.18, and 3.97 mm to simulate the steel spheres with the same size in a 3D DEM simple shear test with the specimen height of 28 mm and diameter of 101.6 mm. The specimen height to particle diameter ratio is 11.76, 8.81, and 7.05. In addition, to study the size effect, 60,000 equal number of particles with the diameter of 1.19, 1.59, and 1.98 mm is used in the same setting as compare group. The specimen height to particle diameter ratio is 23.53, 17.61, and 14.14. Results showed that a small number of particles will cause inhomogeneity and sample boundary effects, which will affect experimental and numerical results.

ASTM D6528, Standard Test Method for Consolidated Undrained Direct Simple Shear Testing of Fine Grain Soils, specifies the specimen height to maximum particle diameter ratio in a consolidated undrained direct simple shear test, which should be greater than 10. It can be seen that to achieve calculation efficiency, some previous studies violate the requirement for minimum specimen height to maximum particle diameter ratio. In addition, the ASTM D6528 also specifies the test diameter to height ratio in a consolidated undrained direct simple shear test, which should be greater than 2.5, some previous DEM studies also violate the requirement for minimum diameter to height ratio. This may cause strong boundary effects and inaccurate results. This ratio is an empirical conclusion drawn through many experimental tests in which have many uncontrollable factors. Therefore, it is important to note the size effect within a DEM simple shear test, and its effect on macro and meso-mechanical behaviour.

## Material and methods

**Figure 1:**
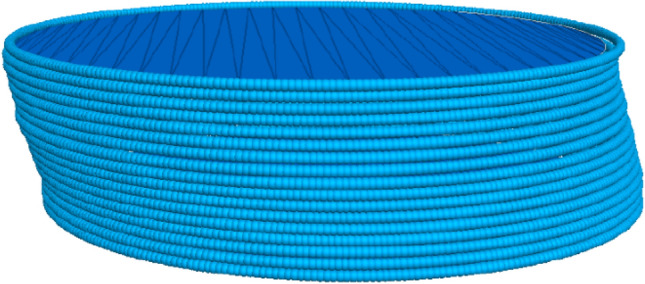
Boundary condition of the DEM model.

Particle Flow Code in three dimensions (PFC 3D) which is based on the discrete element method is used in this study. Soil particles are modeled as rigid spheres (referred to as balls). The contacts between balls are modeled using the soft contacts approach that allows a particle to virtually overlap. In previous studies, the boundary of a particulate DEM model consists of the top wall, bottom wall, and layers of cylindrical sidewalls, which represent the top cap, bottom pedestal, and ring-shape side boundary, respectively^4^. However, in PFC 3D, the rigid cylindrical sidewall cannot be passively moved by particles, the lateral ring-shape wall is controlled by fixed velocities at a different height. As a result, a controlled moving wall causes extra load concentration near the boundary of a specimen. In this study, rings modeled by clumps are used as the boundary of the particulate DEM model which can provide an accurate boundary condition, and is effective in computation since no contact force is needed for the clump, the boundaries are shown in Fig. 1. The DEM model used in this numerical simulation is run by a 12-core processor, even the model of a sample with the largest number of particles can be completed within 8 hours, and this is achieved by the more effective boundary condition.

**Figure 2:**
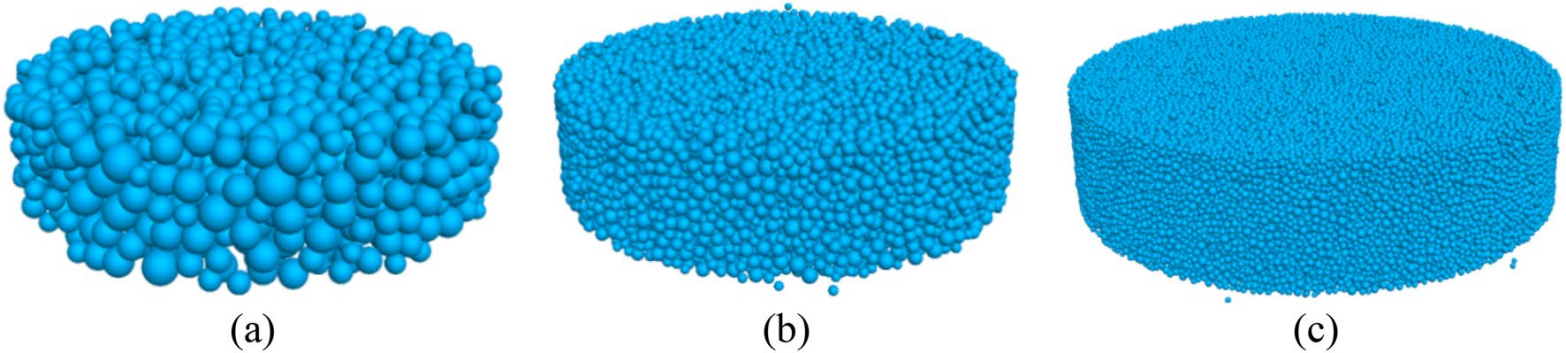
Samples with different particle diameters: (**a**) 4D, (**b**) 2D, (**c**) 1D.

In this study, DEM models are developed according to the bi-directional simple shear test. The simulated sand is Leighton Buzzard sand (Fraction B), its diameter ranges from 0.6 to1.2 mm with a mean value of 0.82 mm, and the effective grain size is 0.65 mm with a uniformity coefficient of 1.38. The particle diameter in DEM is adjusted accordingly in different groups of tests. Leighton Buzzard sand is a sub-rounded spheroid with a relatively smooth surface (3D sphericity mean value 0.92, 3D roundness mean value 0.65, 3D fractal dimension mean value:2.24, elongation index:0.80, flatness index:0.78, convexity:0.91)^8,9^ More details about the testing material and apparatus are introduced by Li et al.^10^. Three groups of tests are simulated using DEM, one with the sand particle size (Noted by 1D model), one with twofold sand particle size (Noted by 2D model), one with fourfold sand particle size (Noted by 4D model). Particles are generated by the radius expansion method with the porosity of 0.37 in a cylinder with 70 mm diameter and 22 mm height which is the same size in the laboratory specimen. Firstly, particles at half of their diameters are randomly generated in the cavity enclosed by clumps and walls, and then expanded to their target sizes. In the DEM test with the particle diameter same to sand, 111,662 balls are generated, 14,061 balls are generated in the test with twofold sand particle size, and 1751 balls are generated in the test with fourfold sand particle size, as shown in Fig. 2. For the 1D, 2D, and 4D models, specimen height to maximum particle diameter is 14.17, 7.08, and 3.54, respectively. In this study, a ball-facet model is used to represent the contact between particles and loading walls (top plate and bottom plate), and a ball-pebble model is used to represent the contact between particles and clump rings.

Many previous studies used the linear contact model in simulations^11–13^, which leads to excessive rolling compared with real granular materials. Qian et al.^14^ indicated that the omission of particle rolling tends to underestimate the shear stress level and overestimate the post-peak dilatation, compared with experimental observations^1^. Rolling is the dominant meso-deformation mechanism controlling the strength and dilatation of granular soils. To avoid interference from other uncontrollable factors, a rolling resistance linear contact model is adopted. Compared with the linear contact model, the rolling resistance linear contact model incorporates a rolling contact component in addition to the normal and tangential contact components.

In the numerical simulation, the input contact parameters will affect the macroscopic and mesoscopic behavior of sand particles^15–18^. DEM models in this study are calibrated using bi-directional simple shear test data presented by Li et al.^10^. A good agreement degree of stress–strain behavior between test and DEM models indicates a satisfactory calibration. Calibration is divided into four stages: (1) calibration of effective modulus which mainly controls the slope stress–strain curve; (2) calibration of friction coefficient which mainly controls the peak shear stress value; (3) calibration of rolling resistance coefficient which also controls the peak shear stress value; (4) minor adjustment of friction coefficient and rolling resistance coefficient for getting a better fitting curve.

The main contact parameters after calibration are listed in Table 1. Parameters for Ball-Facet are identical in all tests, with an effective modulus of 2 × 10^7^(N/m^2^), a friction coefficient of 0.9, and a rolling resistance coefficient of 0.9. The two high coefficients are used for simulating the experimental high friction between the top/bottom load cell and a specimen. Parameters for Ball-Pebble are identical in all tests, with an effective modulus of 2 × 10^7^(N/m^2^), a friction coefficient of 0.1, and a rolling resistance coefficient of 0.1. The two low coefficients are used for simulating the low friction between the latex membrane and a specimen. Parameters for Ball-Ball are the main focus of the calibration, the effective modulus, friction coefficient, and rolling resistance coefficient are: 5.3 × 10^7^(N/m^2^), 0.45, 0.18 respectively in the 1D test; 4.4 × 10^7^(N/m^2^), 0.5, 0.17 respectively in the 2D test; 1.2 × 10^7^(N/m^2^), 0.5, 0.4 respectively in the 4D test. The stiffness ratio is 1 for all contacts, The density of particles in DEM is 2650 which corresponds to the Specific Gravity (G_s_) of 2.65 for the Leighton Buzzard Sand Fraction B.

**Table 1:** Summary of calibrated contact model parameters.

Group name	Contact type	Effective modulus (N/m^2^)	Friction coefficient	Rolling resistance coefficient
1D	Ball–ball	5.3 × 10^7^	0.45	0.18
	Ball–facet	2 × 10^7^	0.9	0.9
	Ball–pebble	2 × 10^7^	0.1	0.1
2D	Ball–ball	4.4 × 10^7^	0.5	0.17
	Ball–facet	2 × 10^7^	0.9	0.9
	Ball–pebble	2 × 10^7^	0.1	0.1
4D	Ball–ball	1.2 × 10^7^	0.5	0.4
	Ball–facet	2 × 10^7^	0.9	0.9
	Ball–pebble	2 × 10^7^	0.1	0.1

**Figure 3:**
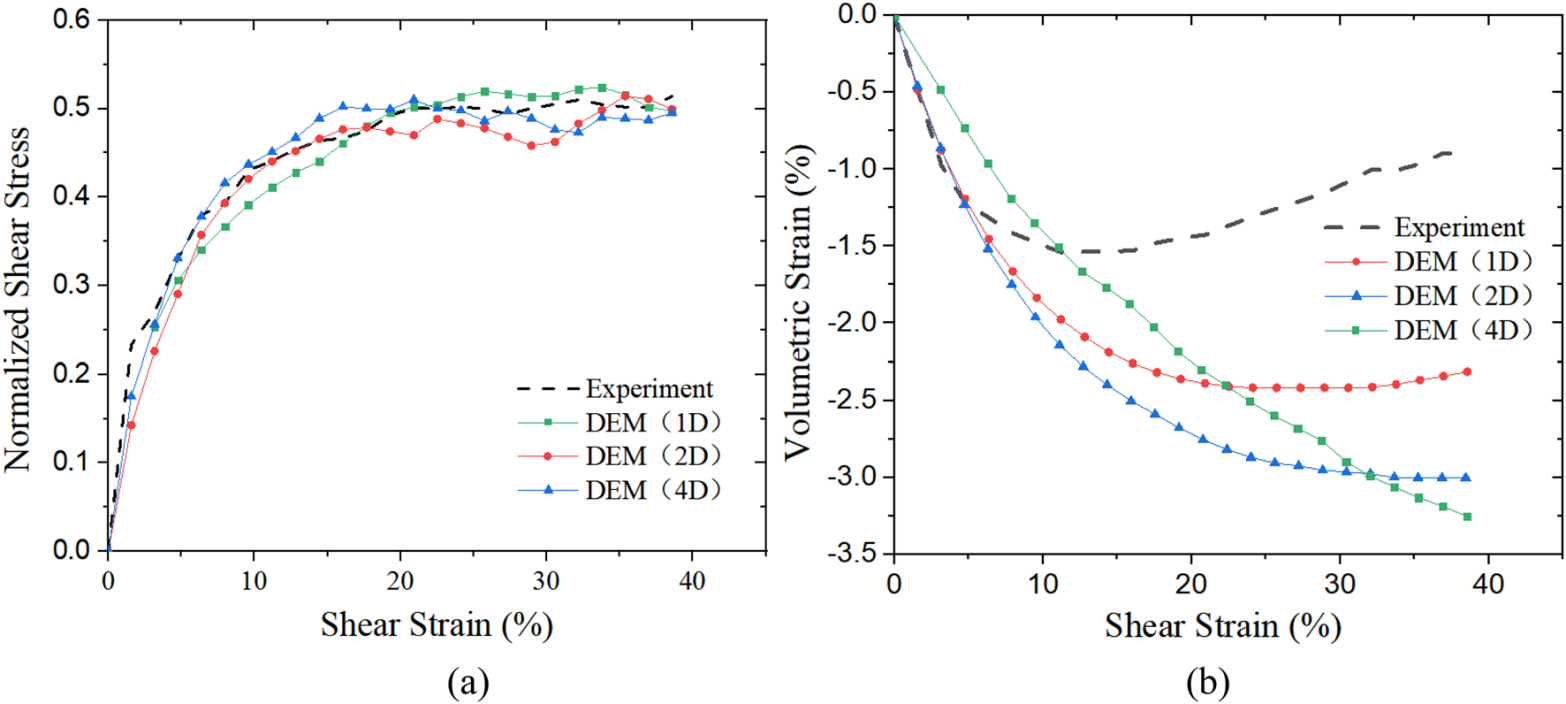
Macro-mechanical behavior in DEM models: (**a**) Shear stress and shear strain relations (**b**) Shear strain and volumetric strain relations.

Figure 3a shows the shear stress and shear strain relations in the simple shear test and DEM tests with different particle diameters. By comparing the four curves, it can be found that with the increase of shear strain, the shear stress value fluctuates in a small range, but the general trend is similar. Figure 3b shows the volumetric strain response with shear strain. With the increase of shear strain, volumetric strain in 1D and 2D models gradually increase, and then decrease slowly after reaching the peak value. Modeled shear strain and volumetric strain relation are similar to that in the laboratory tests indicating the DEM model is well calibrated. In the 4D test, volumetric strain gradually increases without decreasing which is different from laboratory tests indicating the DEM model is not ideal.

Relative differences (the difference between the experimental value and modeled value divided by experimental value) of the three tests from the laboratory test results are presented in Table 2, which includes the maximum and average values of the relative difference in shear stress and volumetric strain, respectively. The relative differences indicated that the 3 models provide a good simulation of experimental stress–strain behavior, but the volumetric strain behavior is not ideal. Generally, the 1D model provides the best fit for the stress–strain and volumetric strain behavior. It should be noted, the stress–strain relation is the main focus of calibration, while the volumetric strain relation is used as a minor factor. This is due to the fact that it is hard to satisfy the two relations simultaneously due to the simplified particle shape and increased diameters in DEM models.

**Table 2:** Summary of the relative differences in shear stress and volumetric strain.

Model name	Maximum of relative difference in shear stress (%)	Mean of the relative difference in shear stress (%)	Maximum of relative difference in volumetric strain (%)	Mean of the relative difference in volumetric strain (%)
1D	10.4	1.46	161.71	67.69
2D	16.3	3.51	235.53	106.08
4D	7.61	0.06	263.46	91.53

## Results

**Figure 4:**
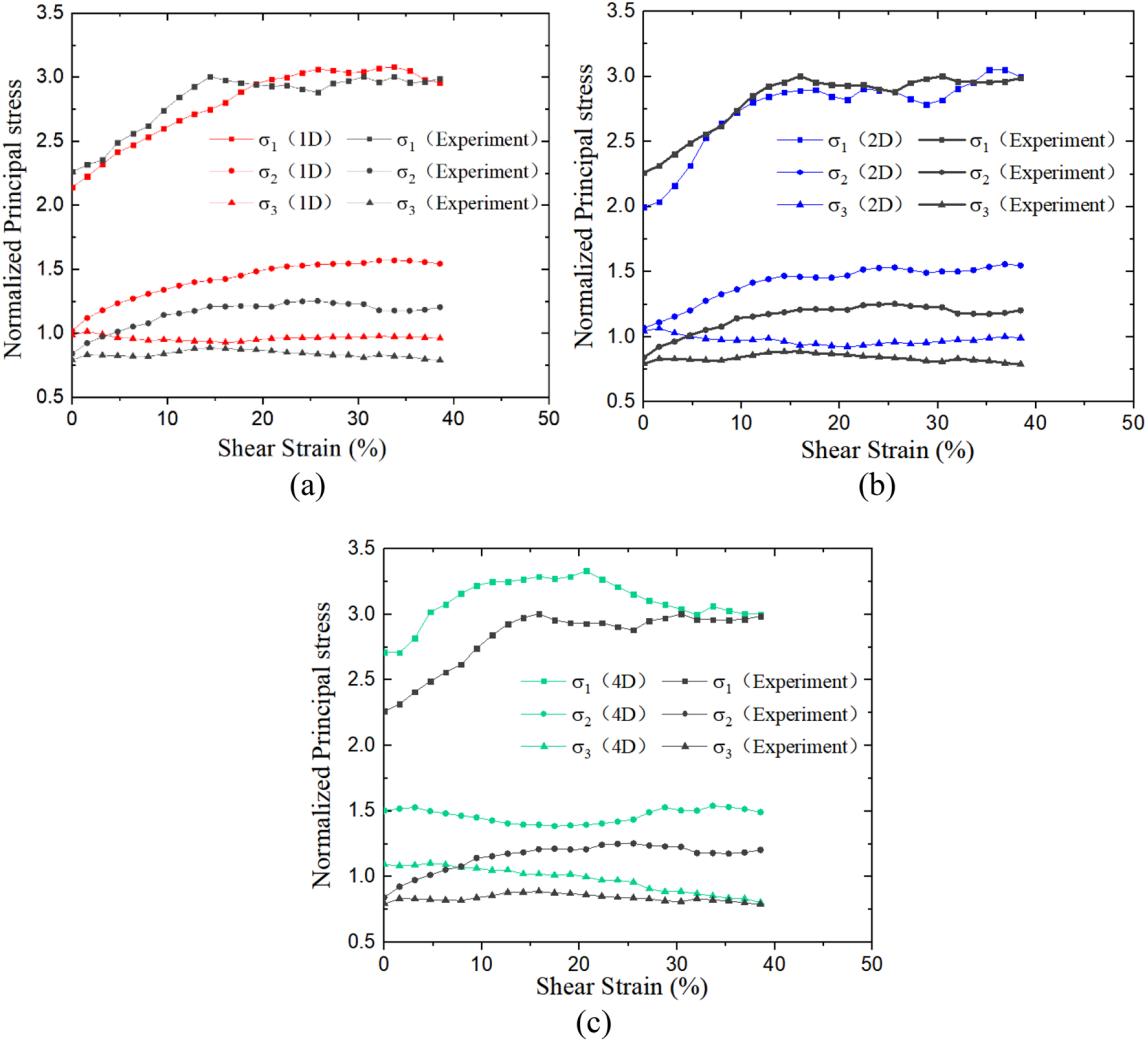
Principal stress and shear strain relations: (**a**) 1D model and Experiment, (**b**) 2D model and Experiment, (**c**) 4D model and Experiment.

**Figure 5:**
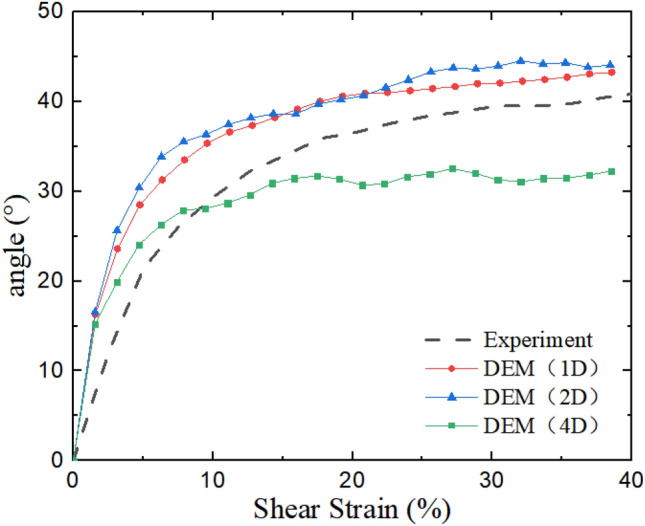
Rotation of principal stresses.

In DEM simulations, the complete stress state can be measured via measurement function, and it is transformed to principal stresses for further analysis, as shown in Fig. 4. The rotation of principal stress is presented in Fig. 5. In Figs. 4 and 5, simulated resulted are compared with laboratory test results determined by a method proposed by Budhu and validated in the previous study^19–21^, this is due to the fact that in simple shear tests horizontal stresses cannot be accurately measured.

Table 3 shows the summary of the relative difference in principal stress, combining with Fig. 4, it can be seen that the stresses in 1D and 2D models are closer to the experimental results, in which the major principal stresses have a mean relative difference smaller than 4% while it is 10.59% in the 4D model. Compared with the results of laboratory tests, the principal stresses of the 4D test are larger and its maximum rotation angle of principal stress is only about 30°, while the rotation angle of the principal stress in the 1D and 2D tests eventually stabilized at around 42°, similar to the results reported by Asadzadeh and Soroush^5^, also by Bernhardt et al.^22^.

**Table 3:** Summary of the relative difference in principal stress.

Model name	Principal stress	Maximum of relative difference in principal stress (%)	Mean of the relative difference in principal stress (%)
1D	σ_1_	8.59	3.57
	σ_2_	33.23	23.46
	σ_3_	21.72	15.13
2D	σ_1_	12.06	3.60
	σ_2_	31.55	23.24
	σ_3_	27.98	16.73
4D	σ_1_	21.26	10.59
	σ_2_	78.46	28.86
	σ_3_	37.71	17.28

It should be noted even the stresses in the 1D model have certain differences compared with experimental results, this is mainly due to the different measuring mechanics in experimental tests and DEM models. In DEM models, a measurement ball is used in the center of a specimen, and the presented values are calculated using standard mechanical equations based on the measured values with minor boundary effects. In experimental tests, the presented values are calculated based on boundary measurements (vertical normal stresses and shear stresses) with a strong boundary effect. In addition, the stress determination method proposed by Budhu^20^ is based on the peak shear stress state which neglects the effect of K_0_ consolidation and causes the difference at the initial shear stage. According to Table 3, it can be seen that the relative differences of major principal stress are much lower than others, this is due to the fact that at the initial shear stage the major principal stress equals vertical normal stress, and is not affected by K0 consolidation.

**Figure 6:**
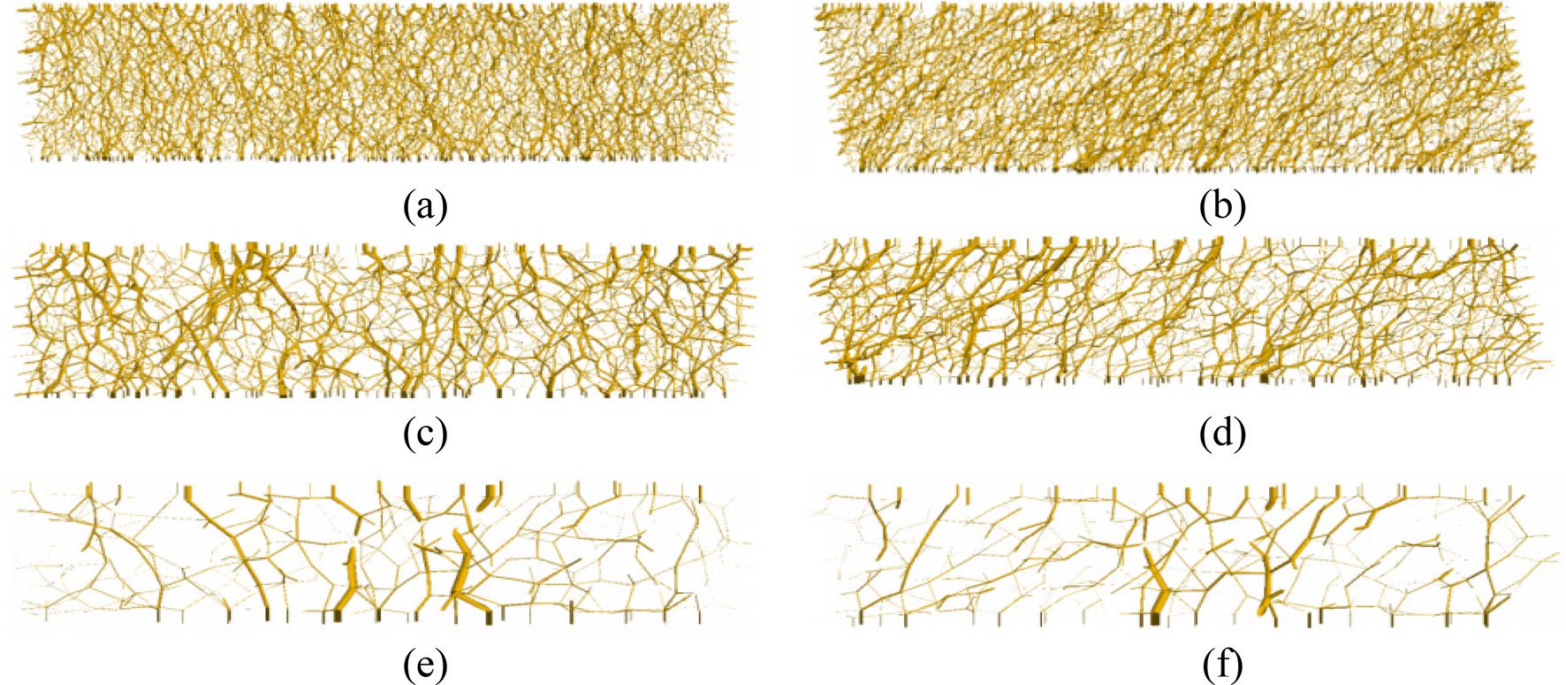
Contact force networks: (**a**) 1D after consolidation, (b) 1D 20% shear strain, (**c**) 2D after consolidation, (**d**) 2D 20% shear strain, (**e**) 4D after consolidation, (**f**) 4D 20% shear strain.

Figure 6 shows the contact forces at a block with y =  − 0.002 m to y = 0.002 m (along shear direction) during different stages of simulations, the thickness of the lines in the figures indicates the magnitude of contact forces and the orientation of the lines denotes the direction of contact forces. Figure 6a,c,e show the contact force network after consolidation under 200 kPa. In 1D and 2D models, large contact forces are uniformly distributed along the vertical direction which is also the direction of major principal stress. In the 4D model, the number of contact forces in the contact force network diagram is relatively small, and the distribution of contact force is nonuniform indicating the specimen is inhomogeneous. As a result, there is a large stress concentration in the 4D model, but the phenomenon hardly occurs in 1D and 2D models. Figure 6b,d,f show that the direction of contact force rotated to 45° at 20% shear strain, this phenomenon is also reported by Asadzadeh and Soroush^5^. This is due to the fact that principal stresses rotate during shear, which causes the contact force to rotate in the same direction^23–25^.

**Figure 7:**
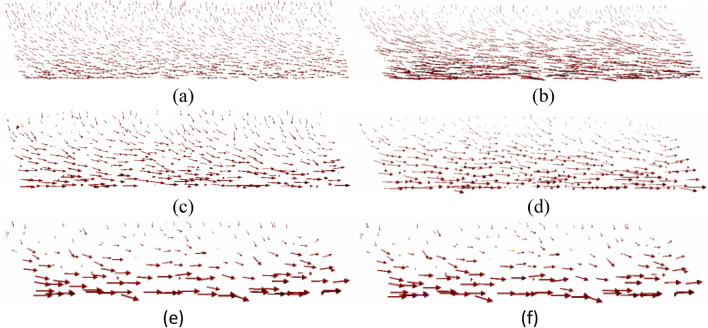
Incremental displacement vectors for global shear strain interval (Front view): (**a**)1D 20% shear strain, (**b**) 1D 40% shear strain, (**c**) 2D 20% shear strain, (**d**) 2D 40% shear strain, (**e**) 4D 20% shear strain, (**f**) 4D 40% shear strain.

**Figure 8:**
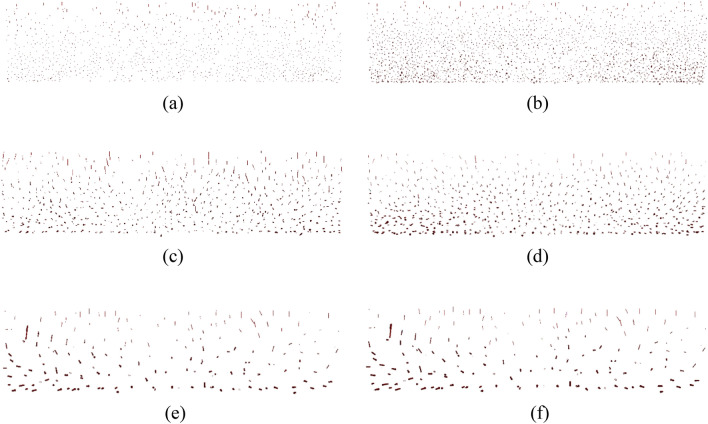
Incremental displacement vectors for global shear strain interval (Side view): (**a**) 1D 20% shear strain, (**b**) 1D 40% shear strain, (**c**) 2D 20% shear strain, (**d**) 2D 40% shear strain, (**e**) 4D 20% shear strain, (**f**) 4D 40% shear strain.

Two orthogonal views of the particle displacements are illustrated in Figs. 7 and 8. Referring first to Fig. 7, for all tests, the displacement direction of the upper particles is generally downward, and their displacement distance is shorter than that of the bottom particles. From the middle to the bottom of specimens, the displacement direction of the particles gradually rotates to the shear direction during shear. However, in the 4D model, two downward displacement vectors can be noted indicating the discontinues and unpredictable movement of 4D particles. Comparing Figs. 7 and 8, it is clear that the components of the particle displacement vectors in the y-direction are smaller than the x-direction which is the shearing direction, this is also reported by Cui and O'sullivan^26^. Vectors in samples with larger diameters are usually larger, and prone to have stress/strain concentration, for example: in Fig. 8 only the 4D test has a large movement in the z-direction (upward). A similar observation was reported by Masson and Martinez^27^.

**Figure 9:**
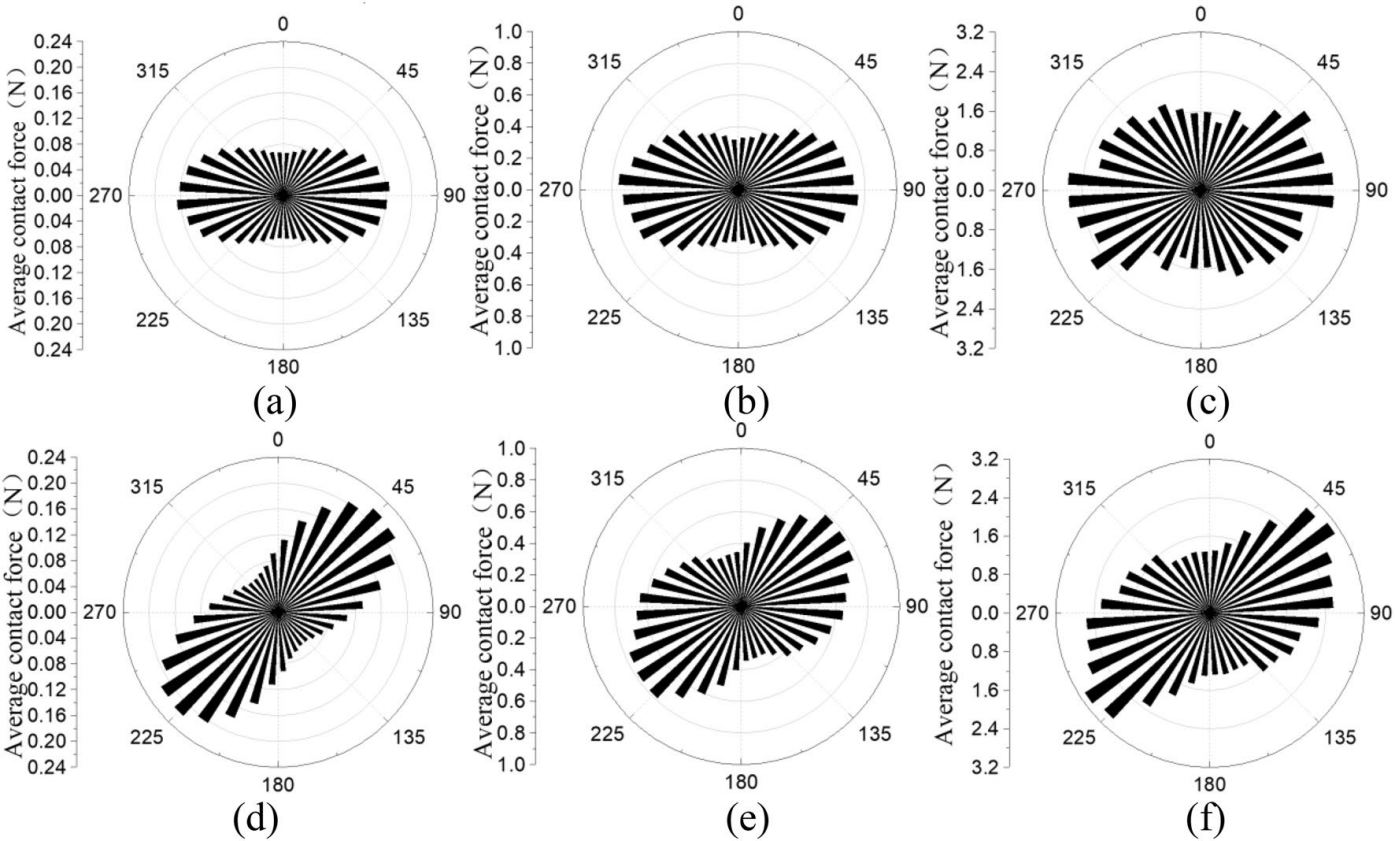
Distribution of average contact force: (**a**) 1D after consolidation, (**b**) 2D after consolidation, (**c**) 4D after consolidation, (**d**) 1D at 40% shear strain, (**e**) 2D at 40% shear strain, (**f**) 4D at 40% shear strain.

Figure 9 shows the qualitative distribution of average contact force after consolidation and at 40% shear strain. A bin shows the magnitude of average contact force increases by 10° in the rose diagram. It is clearly indicated that after consolidation, large contact forces are mainly near 90°. It can be seen that the image of the 4D model is asymmetrical, and the maximum average contact force is around 50° to 100° to horizontal, while it is 90° in 1D and 2D models, this is caused by nonuniformly distributed contact force in the inhomogeneous specimen in 4D model. At 40% shear strain, the maximum contact force is observed at 50° to 60° to horizontal in the 4D model, while it is 40° to 50° in 1D and 2D models. The observed behavior agrees qualitatively with that reported by Asadzadeh and Soroush^5^, and O'Sullivan et al.^28^.

**Figure 10:**
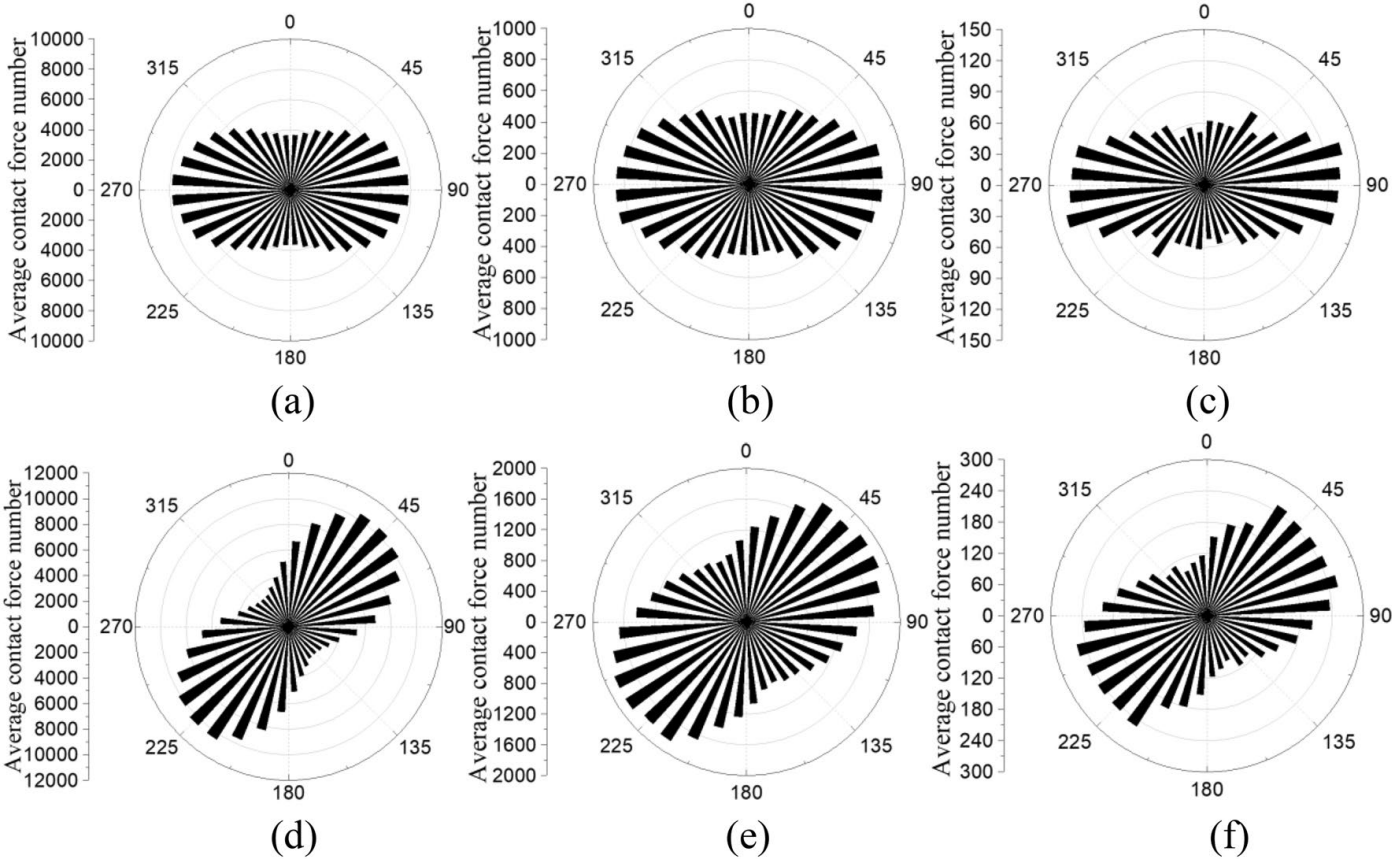
Distribution of average contact number: (**a**) 1D after consolidation, (**b**) 2D after consolidation, (**c**) 4D after consolidation, (**d**) 1D at 40% shear strain, (**e**) 2D at 40% shear strain, (**f**) 4D at 40% shear strain.

Figure 10 shows the qualitative distribution of average contact number after consolidation and at 40% shear strain. After consolidation, the average contact number is nonuniformly distributed in the 4D model, and uniformly distributed in the 1D and 2D models. At 40% shear strain, the average contact number is relatively uniformly distributed in all tests. The direction of majority contact forces is around 45°, tests with smaller particle size have a narrower range, for example, majority contact forces is around 30° to 60° to horizontal in 1D test, around 30° to 70° to horizontal in 2D model, around 30° to 80° to horizontal in 4D model. In the 4D model, the number of contact forces is limited, and the contact forces on each particle are larger (indicated in Fig. 10c,f) which may lead to non-uniform forces and stress concentration. Compared with the number of contact forces after consolidation, the number at 40% shear strain is significantly larger indicating the densification (indicated in Fig. 3b) and the generation of shear-related contacts.

Table 4 summarized the contact numbers and average contact forces at consolidation and 40% shear strain. It can be concluded that increasing particle size increases the average contact force and decreases the contact number, during shear the change of average contact force is limited while the contact number is dramatically increased. In the 1D model, the number of contact forces at 40% shear strain increased 13%, the magnitude of average contact forces increased 18%. In the 2D model, the number of contact forces at 40% shear strain increased 115%, the magnitude of average contact forces increased 6%. In the 4D model, the number of contact forces at 40% shear strain increased 108%, the magnitude of average contact forces decreased  4%. This indicated that during shear, increasing particle size has a complex effect on the increasing rate of contact number, and increasing particle size decreases the increasing rate of average contact forces.

**Table 4:** Summary of the distribution of contact forces.

State	Average contact force (N)			Contact number		
	1D	2D	4D	1D	2D	4D
Consolidation	0.11	0.52	2.04	2.02 × 10^5^	2.30 × 10^4^	3.09 × 10^3^
40% shear strain	0.13	0.55	1.97	2.28 × 10^5^	4.95 × 10^4^	6.42 × 10^3^

The above results show that the 2D model can be used in the simulation of a simple shear test on Leighton Buzzards sand (Fraction B) with less computational effort. The finding partially supports the statement that the 2–2.5 times of the simulated sand diameter could provide reasonable DEM simulation results^2–4^, and reveals a strong stress concentration and specimen inhomogeneity reported in previous studies^6,7^. However, the 2D model has a specimen height to maximum particle diameter ratio of 7 which is smaller than the requirement in ASTM D6528, Standard Test Method for Consolidated Undrained Direct Simple Shear Testing of Fine Grain Soils. The ASTM D6528 specifies that the specimen height to maximum particle diameter ratio should be greater than 10. This is due to the fact that the DEM model can effectively reduce external interference, including inevitable instrument errors, operating errors, calculation errors, small differences in sample size, and particle arrangement changes. In addition, it can avoid some uncontrollable factors in the laboratory tests, making particle size the only variable in the simulation, while there are more variables in a laboratory test.

## Conclusion

In this study, a 3D DEM simple shear model based on the NGI-type bi-directional simple shear test on sand is used to study the size effect of sand particles on macro and meso-mechanical behavior. The DEM model used clump rings to simulate physical rings in the test, and particles can move freely without applied additional forces from side walls. Tests with particle size equal sand (1D model), twofold sand particle size (2D model), and fourfold sand particle size (4D model) are first calibrated using experimental shear stress-shear strain relations, then the size effect is analyzed. Results are summarized as follow:

1. The 4D model is sensitive to subtle changes in assembly, and it has a more dilative behavior and a smaller degree of noncoaxiality compared with that in a laboratory test. 1D and 2D models can better capture the tested stress–strain behavior, volumetric changes, and noncoaxiality.
2. The 4D model has an asymmetrical distribution of contact force and contact number, indicating the specimen is inhomogeneous and has a strong stress concentration.
3. In the distribution of average contact force and average contact number, the image of 4D model is asymmetrical and the maximum value exists in a wider angle range. Increasing particle size has a complex effect on the increasing rate of contact force number, and increasing particle size decreases the increasing rate of average contact forces.

Generally, this study indicated that a test with twofold sand particle size can be used in the simulation of a simple shear test on Leighton Buzzards sand (Fraction B), which has a specimen height to maximum particle diameter ratio of 7. The ratio is smaller than the requirement in ASTM D6528 where it should be greater than 10. In the DEM simulation of a geotechnical test, corresponding requirement in standards should be considered, but the values given in standards can be optimized.
